# Identification of differentially expressed genes in mouse paraspinal muscle in response to microgravity

**DOI:** 10.3389/fendo.2022.1020743

**Published:** 2022-10-13

**Authors:** Yongjin Li, Chao Kong, Baobao Wang, Wenzhi Sun, Xiaolong Chen, Weiguo Zhu, Junzhe Ding, Shibao Lu

**Affiliations:** ^1^ Department of Orthopedics, Xuanwu Hospital, Capital Medical University, Beijing, China; ^2^ National Clinical Research Center for Geriatric Diseases, Xuanwu Hospital, Capital Medical University, Beijing, China

**Keywords:** paraspinal muscle, differentially expressed genes, microgravity, muscle atrophy, paraspinal muscle degeneration

## Abstract

Lower back pain (LBP) is the primary reason leading to dyskinesia in patients, which can be experienced by people of all ages. Increasing evidence have revealed that paraspinal muscle (PSM) degeneration (PSMD) is a causative contributor to LBP. Current research revealed that fatty infiltration, tissue fibrosis, and muscle atrophy are the characteristic pathological alterations of PSMD, and muscle atrophy is associated with abnormally elevated oxidative stress, reactive oxygen species (ROS) and inflammation. Interestingly, microgravity can induce PSMD and LBP. However, studies on the molecular mechanism of microgravity in the induction of PSMD are strongly limited. This study identified 23 differentially expressed genes (DEGs) in the PSM (longissimus dorsi) of mice which were flown aboard the Bion M1 biosatellite in microgravity by bioinformatics analysis. Then, we performed protein–protein interaction, Gene Ontology function, and Kyoto Encyclopedia of Genes and Genomes pathway enrichment analysis for the DEGs. We found that Il6ra, Tnfaip2, Myo5a, Sesn1, Lcn2, Lrg1, and Pik3r1 were inflammatory genes; Fbox32, Cdkn1a, Sesn1, and Mafb were associated with muscle atrophy; Cdkn1a, Sesn1, Lcn2, and Net1 were associated with ROS; and Sesn1 and Net1 were linked to oxidative stress. Furthermore, Lcn2, Fbxo32, Cdkn1a, Pik3r1, Sesn1, Net1, Il6ra, Myo5a, Lrg1, and Pfkfb3 were remarkably upregulated, whereas Tnfaip2 and Mafb were remarkably downregulated in PSMD, suggesting that they might play a significant role in regulating the occurrence and development of PSMD. These findings provide theoretical basis and therapeutic targets for the treatment of PSMD.

## Introduction

Lower back pain (LBP), including nociceptive, neuropathic, nociplastic, or non-specific pain, is one of the most common musculoskeletal disorders and might be experienced by people of all ages (1–3). As a global public health problem, LBP is known to impose a huge socioeconomic burden worldwide each year (3, 4). The maintenance of spinal stability depends on the coordinated movement of the active subsystem [paraspinal muscles (PSM) and tendons], the passive subsystem (vertebrae, intervertebral discs, and ligaments), and the nervous system (5, 6). The structure and the function disorder of any of the above components might contribute to LBP. The psoas major, multifidus, and erector spinae are the most important muscle groups of the PSM, which exert a critical role in maintaining the upright posture and the sagittal balance of the spine (5, 7, 8). In recent years, increasing evidence have revealed that paraspinal muscle (PSM) degeneration (PSMD) is closely correlated with LBP and spine degenerative diseases, such as intervertebral disc degeneration and scoliosis (9–13). Microgravity is caused by factors such as the residual atmosphere in space, which is reported to affect the metabolism of human muscles and bones by regulating the gene expression and the molecular signaling pathways. The space environment is a microgravity environment; this environment can induce PSMD and intervertebral disc degeneration. Thus, microgravity is also a key contributor to LBP (14–16). Nevertheless, the role and the molecular regulatory mechanisms of microgravity in the induction of PSMD remain largely unknown.

Up to now, most studies focused on multifidus degeneration and spine pathophysiology (9, 10, 17–20). Notably, the degeneration of the erector spinae usually occurs earlier, and the degree of degeneration is more serious than that of the multifidus (5, 21). The erector spinae consists of the iliocostalis lumborum, longissimus dorsi, and spinous muscles. The developing fatty infiltration, tissue fibrosis, and muscle atrophy have long been considered as characteristic pathological alterations of PSMD (9). Muscle atrophy is associated with abnormally elevated oxidative stress, reactive oxygen species (ROS), and inflammation (15, 22). Altered gene expression appears to be a hallmark pathological feature of PSMD. Yang et al. (23) found that the serum CXC chemokine ligand 10 concentrations were significantly increased in patients with PSMD compared with patients without PSMD. Kudo et al. (19) observed that anti-muscular dystrophy gene peroxisome proliferator-activated receptor gamma coactivator 1 alpha (PGC-1α) as well as pro-inflammatory cytokines TNF‐α and IL-6 were significantly increased in multifidus from patients with lumbar kyphosis (a reduced lumbar lordosis) compared with normal lumbar lordosis. The genes of the PSM were also differentially expressed in microgravity. Yamakuchi et al. (24) found that 42 genes were differentially expressed in the PSM of rats after 14 days of space flight, among which the heat shock protein 70 and t complex polypeptide 1 were increased, whereas myocyte-specific enhancer binding factor 2C, aldolase A, and muscle ankyrin were decreased. Mirzoev et al. (25) found that ubiquitin ligase MURF-1 was upregulated in the longissimus dorsi of mice after a flight of 30 days. Ogneva et al. (26) found that the content of α-actinin-1/4 and β-actin, respectively, were decreased in the skeletal muscle of mice after a space flight on board the BION-M1 biosatellite for 30 days. However, the effects of microgravity on the gene expression of the longissimus dorsi (erector spinae) remain unclear.

Gambler and colleagues studied the global gene expression profile in the longissimus dorsi of C57BL/N6 male mice after a space flight of 30 days and reported that several genes were associated with insulin resistance and PSM metabolism (27). In this study, we re-analyzed this microarray dataset (GSE94381) downloaded from the public open Gene Expression Omnibus (GEO) database (http://www.ncbi.nlm.nih.gov/geo) (28). R software was used to identify the differentially expressed genes (DEGs). Gene Ontology (GO) and Kyoto Encyclopedia of Genes and Genomes (KEGG) enrichment analysis were performed to predict the potential biological functions of the DEGs.

## Materials and methods

### Analysis of the mRNA microarray dataset

The GSE94381 microarray dataset was downloaded from GEO database through R software GEOquery package (29). The probes corresponding to multiple molecules for one probe were removed, and we kept only the probes with the largest signal value when encountering probes corresponding to the same molecule. The data was then standardized using the normalizeBetweenArrays function of the limma package in R software. Limma package was also used to identify the DEGs according to the following criteria: -log_10_ (*P*-value) > log_10_ 20 and |log_2_ fold-change (FC)| > 1. The Ggplot2 package in R software was used to analyze and visualize the clustering situation between the different groups (30). The Ggplot2 package was also used to visualize the principal component analysis (PCA), uniform manifold approximation and projection (UMAP), and volcano plots. The heat map was visualized using the complexheatmap package (31).

### Venn diagram analysis

In the GSE94381 microarray dataset, the mice were divided into three groups: Bion-flown (BF), Bion ground (BG), and flight control (FC) group (28). The DEGs between the BG and BF groups, the FC and BF groups, as well as the FC and BG groups were intersected to select overlapping genes using http://www.bioinformatics.com.cn, a free online platform for data analysis.

### GO and KEGG pathway enrichment analysis

The potential biological functions of the DEGs were predicted through GO and KEGG enrichment analysis. The GO database divides the gene functions into three parts: cellular component (CC), molecular function (MF), and biological process (BP). CC is used to describe the location of gene products in cells, such as endoplasmic reticulum or nucleus; MF mostly refers to the functions of individual gene products, such as binding activity or catalytic activity; while BP mostly refers to an orderly biological process with multiple steps, such as cell growth, apoptosis, and signal transduction. The KEGG pathway analysis is a comprehensive database that integrates genomic, chemical, and systemic functional information. KEGG has four major categories and 17 sub-databases, one of which is called the KEGG pathway. The results of the GO and KEGG analyses were visualized using an online tool (http://www.bioinformatics.com.cn).

### Identification of oxidative stress-related genes and ferroptosis-related genes

The ferroptosis-related genes (FRGs) were obtained from the FerrDb V2 database (http://www.zhounan.org/ferrdb/) (32), including ferroptosis driver, suppressor, marker, and unclassified regulator, which is shown in **Supplementary Table S1**. The oxidative stress-related genes (OSRGs) were obtained from the molecular signature database (https://www.gsea-msigdb.org/gsea/msigdb/index.jsp) (33) and are listed in **Supplementary Table S2**.

### Protein–protein interaction network construction

The Search Tool for the Retrieval of Interacting Genes (STRING) database (https://cn.string-db.org/) was widely utilized to assess protein–protein interactions (PPIs) in functional protein association networks (34). Then, we input the DEGs into the multiple protein section of the STRING database and set the organisms as “Mus musculus” to construct the PPI network. The “required score” was set at >0.4. Finally, we generated and downloaded the results of the PPI network analysis.

### Statistical analysis

The expression data of several key genes in the GSE94381 dataset were analyzed and visualized using GraphPad Prism software. The statistical significance between the two groups was compared by an unpaired Student’s *t-*test, whereas the differences among more than two groups were evaluated by one-way analysis of variance followed by Turkey’s multiple-comparisons test. The results were expressed as mean ± standard deviation. *P*-value <0.05 was determined to statistical significance (** represents *p* < 0.01 and *** represents *p* < 0.001).

## Results

### Evaluation of the reasonableness of the sample and grouping

To explore the DEGs that can regulate PSMD in microgravity, we analyzed the microarray dataset GSE94381. The basic information of GSE94381 is shown in **Table 1**. Given that the BF group of mice (experimental group) was flown aboard the Bion M1 biosatellite in microgravity, the authors designed the BG group wherein mice were housed in the same condition but exposed to Earth’s gravity and the FC group wherein mice were housed in a standard animal facility to rule out the effect of housing conditions. We analyzed the transcriptome of the BG–BF group, the FC–BF group, and the FC–BG group, respectively.

**Table 1:** Basic information on GSE94381.

Dataset	Platform	Samples	RNA	Year	Organism	Region
GSE94381	GPL8321	5/5/5	mRNA	2017	*Mus musculus*	China

**Figure 1:**
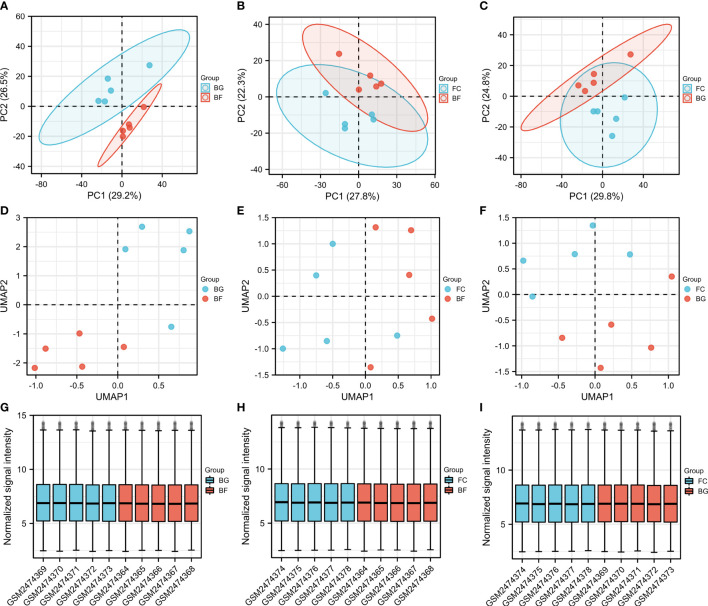
Evaluation of the reasonableness of the sample and grouping. **(A–C)** Principal component analysis diagrams. The horizontal and vertical coordinates show the relative distance among different samples. **(D–F)** Uniform manifold approximation and projection diagrams. **(G–I)** Box plots of samples—normalized. The X‐axis represents samples, and the Y‐axis represents normalized signal intensity.

PCA is a technique for simplifying datasets, which reflects the difference and the distance between different samples by presenting multiple sets of data on the coordinate axis. The closer the distance in the PCA diagram, the more similar the sample composition. As shown in **Figures 1A–C**, the distance between the same group of samples was closer, while the samples between the BG and BF groups, the FC and BF groups, as well as the FC and BG groups were separated, suggesting that the within-group differences were smaller, while the differences between groups were obvious, and there may be more meaningful results in the subsequent difference analysis. The UMAP plot could clearly distinguish the different groups, which further supported the rationality of sample grouping (**Figures 1D–F**). The box plot was utilized to confirm the distribution trends of the hybridization data and the degree of dispersion. As shown in **Figures 1G–I**, we did not observe abnormal distributions of data in the six different samples, revealing that the data have already been standardized.

### Identification of the DEGs

**Figure 2:**
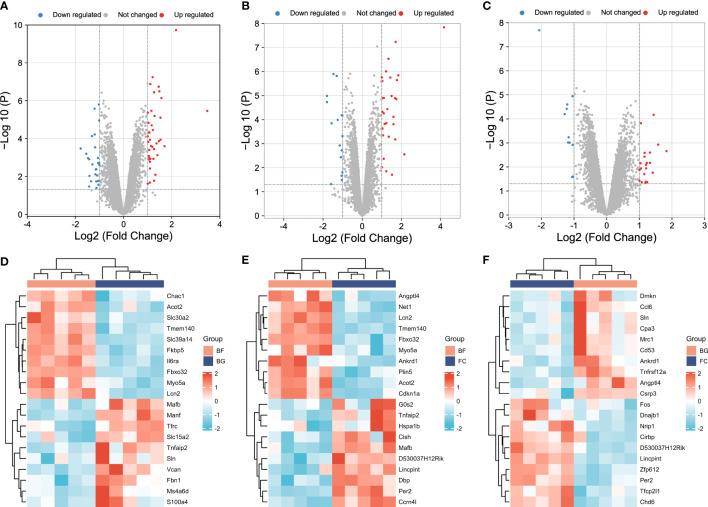
Identification of differentially expressed genes (DEGs) among different groups. **(A–C)** Volcano plots showing the gene expression variation among different groups. **(D–F)** Hierarchical clustering of the first 20 DEGs [-log_10_ (*P*-value) > log_10_ 20]. In the heat map, the red color indicates the upregulated DEGs, and the green color indicates the downregulated DEGs. In the volcano plot, the X‐axis is fold change (log_2_), and the Y‐axis is *P* (−log_10_). The red points indicate the upregulated DEGs, the blue points indicate the downregulated DEGs, and the gray points indicate the genes that are not differentially expressed.

In our study, the thresholds of -log_10_ (*P*-value) > log_10_ 20 and |log2 (FC)| > 1 were used to select the DEGs. The volcano plots were used to evaluate the gene expression variation among different groups. The cluster heat map showed the first 20 DEGs. A total of 27 DEGs were downregulated and 40 DEGs were upregulated between the BG and BF groups (**Figures 2A, D**), 16 DEGs were downregulated and 30 DEGs were upregulated between the FC and BF groups (**Figures 2B, E**), and 11 DEGs were downregulated and 21 DEGs were upregulated between the FC and BG groups (**Figures 2C, F**).

**Figure 3:**
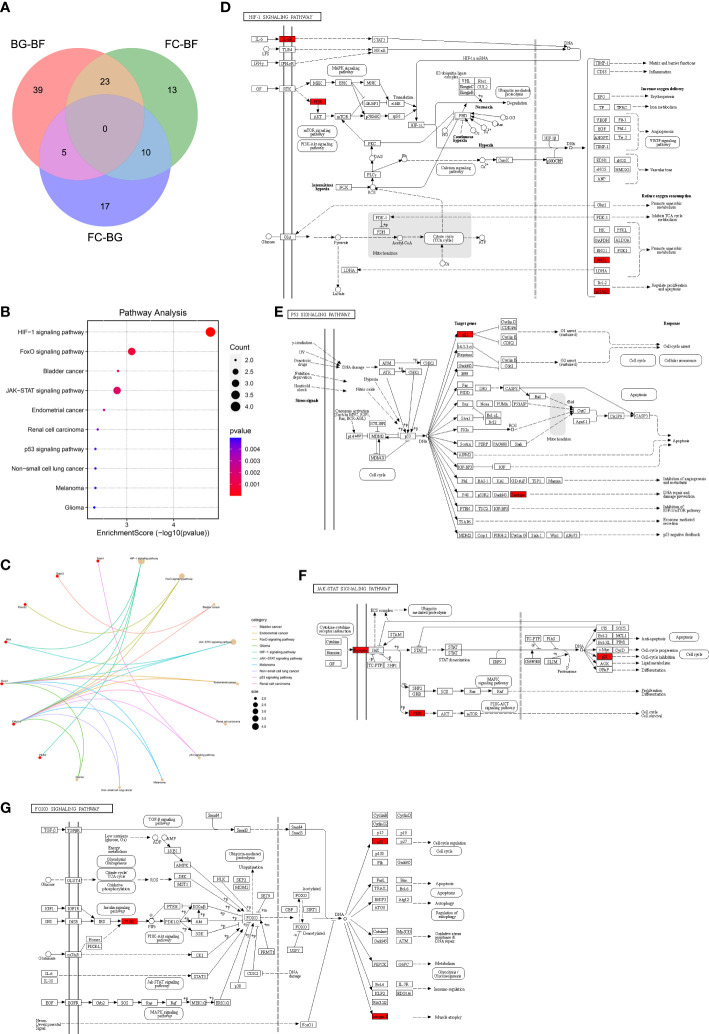
Kyoto Encyclopedia of Genes and Genomes (KEGG) pathway enrichment analysis. **(A)** The 23 overlapping differentially expressed genes (DEGs) between the BG–BF group and the FC–BF group were selected using Venn analysis. **(B, C)** KEGG pathway enrichment analysis of the 23 DEGs. **(D)** KEGG analysis of the HIF signaling pathway. **(E)** KEGG analysis of the p53 signaling pathway. **(F)** KEGG analysis of the JAK-STAT signaling pathway. **(G)** KEGG analysis of the FOXO signaling pathway.

First, a total of 32 DEGs were identified between two control groups. Second, we observed that there were only five overlapping DEGs by intersecting BG–BF and FC–BG as well as 10 DEGs by intersecting FC–BF and FC–BG through Venn analysis (**Figure 3A**), suggesting that the BION-M1 biosatellite, on its own, hardly affected the alteration of gene expression in flown mice muscle compared with the ground control (BG). We also found 23 overlapping DEGs by intersecting BG–BF and FC–BF (**Figure 3A**); then, we determined to study the functions of these DEGs.

### KEGG pathway enrichment analysis

Functional enrichment analysis is critical for elucidating high-throughput omics data in life science. The KEGG pathway enrichment analysis showed that the 23 DEGs were enriched in the HIF-1 signaling pathway, FoxO signaling pathway, bladder cancer, JAK-STAT signaling pathway, endometrial cancer, renal cell carcinoma, p53 signaling pathway, melanoma, non-small cell lung cancer, and glioma (**Figure 3B**). Furthermore, Il6ra, pik3r1, and Cdkn1a were involved in the regulation of JAK-STAT signaling pathway and HIF-1 signaling pathway, thereby mediating cell cycle, proliferation, apoptosis, inflammatory response, *etc.* (**Figures 3C, D, F**). Sesn1 and Cdkn1a might regulate DNA repair and damage prevention and cell cycle arrest through mediating the p53 signaling pathway (**Figures 3C, E**). Additionally, Fbox32, pik3r1, and Cdkn1a were involved in the regulation of FoxO signaling pathway, which can regulate muscle atrophy, cell cycle, *etc.* (**Figures 3C, G**).

### GO functional enrichment analysis

**Figure 4:**
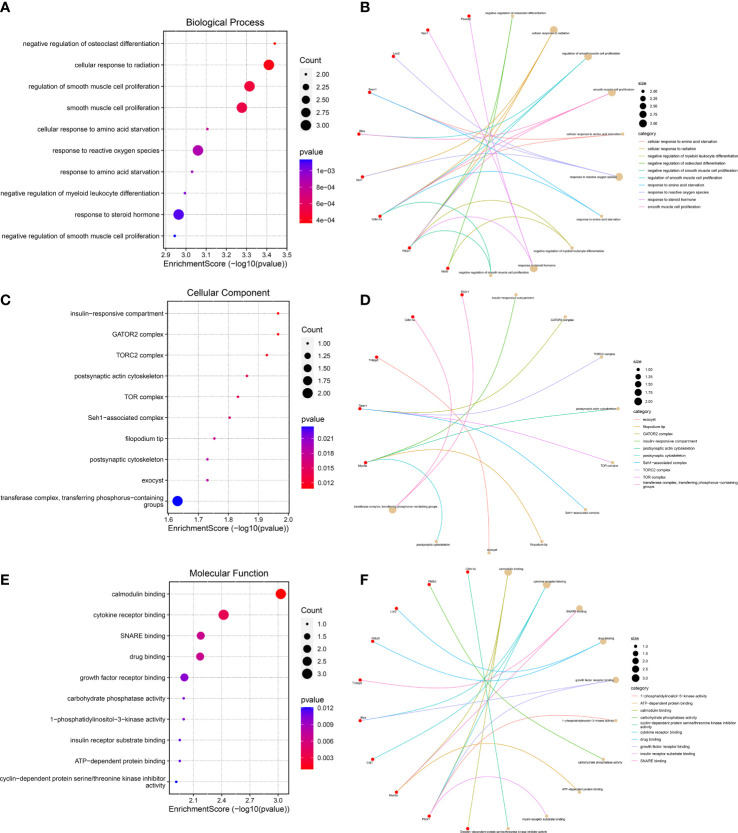
Gene Ontology functional enrichment analysis. **(A, B)** Top 10 biological processes in which the 23 genes may be involved. **(C, D)** The top 10 cellular components were displayed as bubble diagram and cnetplot. **(E, F)** The enrichment of the top 10 molecular functions was displayed by a bubble diagram and cnetplot.

To further predict the functions of DEGs, we conducted GO functional enrichment analysis. GO function annotations included BP, MF, and CC. As shown in **Figures 4A, B**, the top 10 BP terms were enriched in the negative regulation of osteoclast differentiation, cellular response to radiation, regulation of smooth muscle cell proliferation, response to reactive oxygen species, negative regulation of myeloid leukocyte differentiation, response to steroid hormone, *etc.*, of which lipocalin-2 (Lcn2), sestrin 1 (Sesn1), and neuroepithelial cell transforming 1 (Net1) were involved in the regulation of cell response to reactive oxygen species, interleukin 6 receptor alpha (Il6ra), phosphatidylinositol 3-kinase, regulatory subunit polypeptide 1 (Pik3r1), and cyclin-dependent kinase inhibitor p21 (Cdkn1a) were involved in the regulation of smooth muscle cell proliferation, and muscle atrophy F-box32 (Fbxo32), Pik3r1, and Niemann-Pick C1 protein (Npc1) were related to “response to steroid hormone”. Mafb was involved in the negative regulation of osteoclast and myeloid leukocyte differentiation. The CC were predicted to be predominantly enriched in the following terms: insulin-responsive compartment, GATOR2 complex, TORC2 complex, postsynaptic actin cytoskeleton, TOR complex, Seh1-associated complex, filopodium tip, exocyst, postsynaptic cytoskeleton, and transferase complex—transferring phosphorus-containing groups, of which sesn1 was related to “GATOR2 complex, TORC2 complex, TOR complex, and Seh1-associated complex”, and Pik3r1 and Cdkn1a were related to “transferase complex, transferring phosphorus-containing groups” (**Figures 4C, D**). The genes involved in MF are as follows: calmodulin binding, cytokine receptor binding, SNARE binding, growth factor receptor binding, 1-phosphatidylinositol-3-kinase activity, carbohydrate phosphatase activity, insulin receptor substrate binding, and cyclin-dependent protein serine/threonine kinase inhibitor activity—of which Il6ra was involved in cytokine receptor binding and growth factor receptor binding, and Pik3r1 was related to “calmodulin binding, cytokine receptor binding, growth factor receptor binding, 1-phosphatidylinositol-3-kinase activity, and insulin receptor substrate binding” (**Figures 4E, F**).

### Identification of PSMD-related DEGs

**Figure 5:**
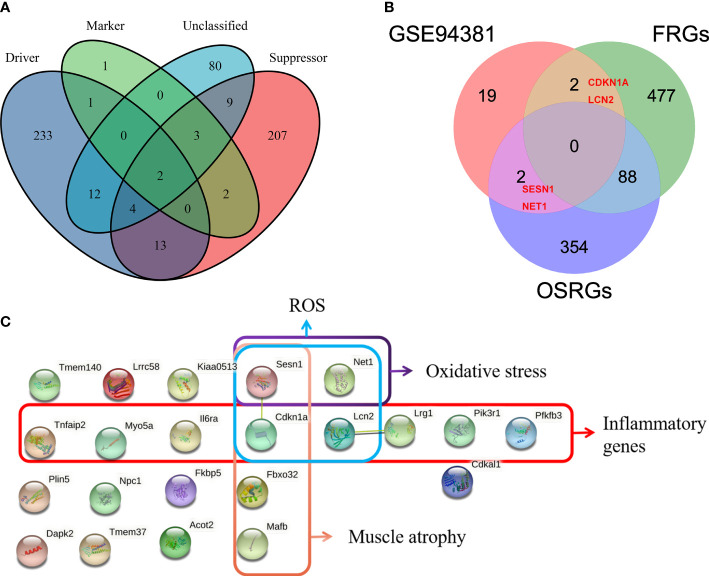
Identification of paraspinal muscles (PSM) degeneration-related differentially expressed genes (DEGs). **(A)** Venn diagram showing that a total of 567 different ferroptosis-related genes (FRGs) were obtained from the FerrDb V2 database. **(B)** Venn analysis was used to identify FRG- and oxidative stress-related genes from 23 DEGs. **(C)** Protein–protein interaction network analysis of the 23 DEGs, of which muscle atrophy-, ROS-, oxidative stress-, and inflammation-related genes were checked.

Given that the abnormally increased oxidative stress, ROS, and inflammation and muscle atrophy are the important pathological mechanism of PSMD, the aim of this study was to explore the relevant genes. Ferroptosis is iron-dependent cell death, and the production of a large number of ROS is its hallmark pathological feature (35). A total of 567 FRGs were identified from the FerrDb database *via* intersecting ferroptosis driver, suppressor, marker, and unclassified regulator (**Figure 5A**). Thus, we merged FRGs, OSRGs, and DEGs to determine the PSMD-related DEGs. The result unveiled that Cdkn1a and Lcn2 were related to ROS and that Sesn1 and Net1 were associated with oxidative stress as well (**Figure 5B**). Using the STRING database, we constructed a PPI network of 23 DEGs and visualized them (**Figure 5C**). Combining literature reports and the results of functional enrichment analysis, we determined that Il6ra, TNF-α-inducible protein 2 (Tnfaip2), myosin Va (Myo5a), Sesn1, Lcn2, leucine-rich α-2 glycoprotein1 (Lrg1), and Pik3r1 were inflammatory genes; Fbox32, Cdkn1a, Sesn1, and musculoaponeurotic fibrosarcoma oncogene (MAF)/MAF family B (Mafb) were associated with muscle atrophy; Cdkn1a, Sesn1, Lcn2, and Net1 were associated with ROS; and Sesn1 and Net1 were linked to oxidative stress (**Figure 5C**).

### The expression of PSMD-related DEGs in GSE94381

**Figure 6:**
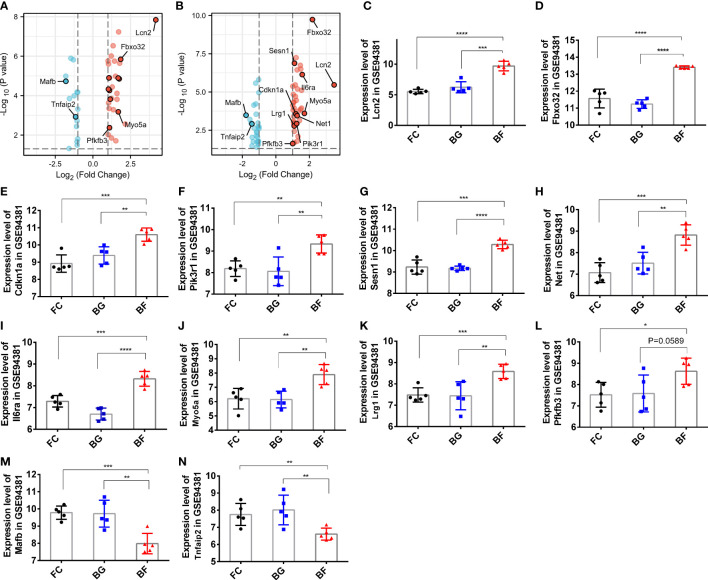
Expression of paraspinal muscles degeneration (PSMD)-related differentially expressed genes (DEGs) in the GSE94381 dataset. **(A)** Volcano plot visualizing the differential expression of PSMD-related DEGs between the flight control (FC) group and the Bion-flown (BF) group in the GSE94381 dataset. **(B)** Volcano plot visualizing the differential expression of PSMD-related DEGs between Bion ground (BG) and BF groups in the GSE94381 dataset. **(C**–**N)** Expression of Lcn2, Fbox32, Cdkn1a, Pik3r1, Sesn1, Net1, Mafb, Il6ra, Myo5a, Tnfaip2, Lrg1, and Pfkfb3 in the FC, BG, and BF groups. * represents p<0.05, ** represents p<0.01 and *** represents p<0.001.

Through the abovementioned analysis, we found that Lcn2, Fbxo32, Cdkn1a, Pik3r1, Sesn1, Net1, Il6ra, Myo5a, Lrg1, and phosphofructo-2-kinase/fructose-2,6- biophosphatase 3 (Pfkfb3), Tnfaip2, and Mafb might play a significant role in regulating the development of longissimus dorsi muscle in mice. Subsequently, we analyzed their expression in different groups. Compared with the FC and BG groups, Lcn2, Fbxo32, Cdkn1a, Pik3r1, Sesn1, Net1, Il6ra, Myo5a, Lrg1, and Pfkfb3 were significantly upregulated, whereas Mafb and Tnfaip2 were significantly downregulated in the BF group (**Figures 6A–N**).

## Discussion

Spinal degeneration and instability are not only due to changes in bone and intervertebral disc structure and function but also to PSMD. The limitation of the current research on the PSM is that they only measure the morphological parameters of PSM on T2-weighted magnetic resonance imaging, including cross-sectional area, fat infiltration rate, *etc.* The understanding of the molecular pathological mechanism of PSMD is very limited. This year, Anderson et al. (36) collected multifidus muscle for biopsy and confirmed that pro-fibrogenic and pro-atrophic genes were upregulated and that anti-fibrogenic and inflammatory genes were downregulated in patients with lumbar spine pathology. Nevertheless, the research on the molecular mechanism of longissimus dorsi muscle is still a blank. Gambler et al. (27) observed moderate signs of longissimus dorsi atrophy in mice after a space flight of 30 days by performing hematoxylin–eosin, immunohistochemical, and immunofluorescence analyses of longissimus dorsi in mice. This study re-analyzed the gene expression profile of the longissimus dorsi of mice exposed to microgravity for 30 days and found that several DEGs were correlated to oxidative stress, ROS, inflammation, and muscle atrophy, which are the pathogenesis of PSMD.

PSM atrophy is a health problem that is currently receiving increasing attention. Identifying the relevant molecular mechanisms and blocking or alleviating PSM atrophy is therefore of utmost importance, yet the mechanisms leading to muscle atrophy are largely unclear. PSM atrophy may be driven by changes in molecules linked to oxidative stress, ROS, and inflammation (9, 15, 22, 37). Growing evidence has revealed that microgravity can alter the expression levels of muscle atrophy-related genes (15, 24–26). To further study the molecular mechanisms of muscle atrophy in response to microgravity, we performed bioinformatics analysis. Muscle-specific ubiquitin E3 ligase Fbox32 (atrogin-1) was reported to be highly expressed in muscle atrophy and inflammation (38). Anderson et al. ( 36) demonstrated that FBXO32 was found to be remarkably increased in multifidus muscle from 59 patients with lumbar spine pathology by quantitative polymerase chain reaction. Our study also predicted that Fbox32 was upregulated under microgravity, which was consistent with Gambler’s result (27). Another E3 ligase muscle atrophy F-Box (MAFb) was downregulated under microgravity (22, 27). Mahmassani et al. (39) demonstrated that the expression of MAFB was downregulated in older adults and associated with the change in leg muscle mass. These findings are consistent with the results of our analysis. Additionally, the cyclin-dependent kinase inhibitor P21 (Cdkn1a) is an inducer of cell cycle arrest and senescence-associated gene, which also serves as a mediator in controlling muscle atrophy (40, 41) and inflammation (42). Kumar et al. (37) demonstrated that microgravity can substantially induce Cdkn1a expression, which is in agreement with our data. Sestrin 1 (Sesn1) was verified to be significantly downregulated in several models of muscle atrophy, including sarcopenia, and protect muscles against aging-induced atrophy (43). However, Sesn1 was significantly upregulated in mouse PSM in response to microgravity, but its roles and mechanism deserve further study. Thus, Sesn1, Cdkn1a, Fbox32, and Mafb might play a pivotal role in muscle atrophy under microgravity.

Chronic muscle inflammation may contribute to the rapid loss of muscle mass, function, and myofibrillar proteins. Our study observed that Il6ra, Pik3r1, and Lrg1 were involved in cytokine receptor binding; Cdkn1a, Il6ra, Pik3r1, and Pfkfb3 were found as well to belong to the HIF-1 signaling pathway *via* GO and KEGG pathway enrichment analysis. Furthermore, Li et al. (44) reported that the inflammatory gene PIK3R1 was remarkably overexpressed in the total T cells in COVID-19 patients. Liu et al. (45) demonstrated that leucine-rich α-2 glycoprotein 1 (LRG1) may prevent renal fibrosis by inhibiting the inflammatory response and pro-fibrotic cytokines. Interleukin (IL)-6 is a common pro-inflammatory cytokine that regulates many inflammatory pathways. IL6RA is an IL6 receptor. Frempah et al. (46) demonstrated that the absence of IL6RA in keratinocytes can promote an inflammatory response. Wang et al. (47) confirmed that the inhibition of PFKFB3 significantly attenuated the inflammatory response in human valve interstitial cell. Faust et al. (42) found that the decrease in Cdkn1a expression paralleled the reduction in cartilage tissue inflammation, suggesting that Cdkn1a may be associated with cartilage inflammation in osteoarthritis. TNFAIP2, a TNF-α-induced protein, which was an inflammatory-responsive molecule for TNF-α and was upregulated in spinal cord ischemia/reperfusion injury patients (48). Additionally, the expression of protease-activated receptors (PAR) is associated with inflammation, and MYO5A is a PAR1-dependent transcript, which has been implicated in the inflammatory process (49). LCN2, an inflammation-related factor with elevated expression in the skeletal muscle of ob/ob mice with sarcopenia, is associated with muscle atrophy-related inflammation and oxidative stress (50). Keping et al. (51) demonstrated that SESN1 inhibited oxidized low-density lipoprotein-induced activation of NK-κB signaling and reduced the expression of pro-inflammatory cytokines in macrophages. Collectively, the abovementioned studies unveiled that Pfkfb3, Il6ra, Tnfaip2, Myo5a, Sesn1, Lcn2, Irg1, and Pik3r1 were inflammatory genes, suggesting that they might exert a key role in the pathogenesis of PSMD.

Other causes of inducing muscle atrophy are oxidative stress and ROS, which were confirmed to promote muscle protein catabolism and inhibit protein synthesis signaling pathways (52–54). In addition to mediating inflammation and muscle atrophy, Cdkn1a was also involved in the regulation of oxidative stress and ROS under microgravity (37). Sesn1 belongs to the family of stress-inducible proteins that regulate oxidative stress and ROS, thus protecting the cells from various stimuli (55). Additionally, Lcn2 was confirmed to promote mitochondrial ROS production and alleviate mitochondrial oxidative phosphorylation in rat primary cardiomyocytes (56). However, whether they mediate PSMD *via* regulating ROS and oxidative stress is an interesting problem which needs future experiments to be verified.

Our study recognizes some limitations. First, the data was downloaded from GEO database, and we did not perform RNA sequencing. Second, we did not perform molecular biology and animal experiments to demonstrate the expression and the roles of the 12 key DEGs in longissimus dorsi muscle in mice. Lastly, there are inherent differences between mice and humans, so the generalization of mouse findings to humans is limited.

## Conclusion

In conclusion, a total of 23 DEGs were observed to be remarkably dysregulated in the PSM of mice in microgravity by bioinformatics analysis. Moreover, we found that Il6ra, Tnfaip2, Myo5a, Sesn1, Lcn2, Lrg1, and Pik3r1 were linked to inflammatory response; Fbox32, Cdkn1a, Sesn1, and Mafb were associated with muscle atrophy; Cdkn1a, Sesn1, Lcn2, and Net1 were associated with ROS; and Sesn1 and Net1 were linked to oxidative stress. Notably, Sesn1 was involved in the regulation of inflammatory response, muscle atrophy, ROS, and oxidative stress. These results suggest that they might be a therapeutic candidate target for PSMD treatment.

## Data availability statement

The original contributions presented in the study are included in the article/**Supplementary Material**. Further inquiries can be directed to the corresponding author.

## Author contributions

SL and YL: conceived and designed this study. YL: wrote the manuscript. SL: critically reviewed and revised the manuscript. CK, BW, and WS: helped with the bioinformatics analysis. WZ, XC, and JD: created the figures and tables. YL is the only first author. All authors contributed to the article and approved the submitted version.

## Funding

This research was funded by the R&D Program of Beijing Municipal Education Commission (KZ/KM/SZ/SM2022100250**) and Beijing Municipal Medical Science Institute—Public Welfare Development Reform Pilot Project (Capital Medical Research no. 2019-2).

## Conflict of interest

The authors declare that the research was conducted in the absence of any commercial or financial relationships that could be construed as a potential conflict of interest.

## Publisher’s note

All claims expressed in this article are solely those of the authors and do not necessarily represent those of their affiliated organizations, or those of the publisher, the editors and the reviewers. Any product that may be evaluated in this article, or claim that may be made by its manufacturer, is not guaranteed or endorsed by the publisher.

## Supplementary material

Supplementary Table 1 Detailed information on ferroptosis-related genes.

Supplementary Table 2 Detailed information on oxidative stress-related genes.

## References

[1] Vos T Lim SS Abbafati C Abbas KM Abbasi M Abbasifard M . Global burden of 369 diseases and injuries in 204 countries and territories, 1990-2019: A systematic analysis for the global burden of disease study 2019. Lancet (London England) (2020) 396(10258):1204–22. doi: 10.1016/s0140-6736(20)30925-9 PMC7567026 33069326

[2] Hartvigsen J Hancock MJ Kongsted A Louw Q Ferreira ML Genevay S . What low back pain is and why we need to pay attention. Lancet (London England) (2018) 391(10137):2356–67. doi: 10.1016/s0140-6736(18)30480-x 29573870

[3] Knezevic NN Candido KD Vlaeyen JWS Van Zundert J Cohen SP . Low back pain. Lancet (London England) (2021) 398(10294):78–92. doi: 10.1016/s0140-6736(21)00733-9 34115979

[4] Dieleman JL Cao J Chapin A Chen C Li Z Liu A . Us health care spending by payer and health condition, 1996-2016. Jama (2020) 323(9):863–84. doi: 10.1001/jama.2020.0734 PMC7054840 32125402

[5] Panjabi MM . The stabilizing system of the spine. part i. function, dysfunction, adaptation, and enhancement. J Spinal Disord (1992) 5(4):383–9. doi: 10.1097/00002517-199212000-00001 1490034

[6] Panjabi MM . The stabilizing system of the spine. part ii. neutral zone and instability hypothesis. J Spinal Disord (1992) 5(4):390–6. doi: 10.1097/00002517-199212000-00002 1490035

[7] Jun HS Kim JH Ahn JH Chang IB Song JH Kim TH . The effect of lumbar spinal muscle on spinal sagittal alignment: Evaluating muscle quantity and quality. Neurosurgery (2016) 79(6):847–55. doi: 10.1227/neu.0000000000001269 27244469

[8] Masaki M Ikezoe T Fukumoto Y Minami S Tsukagoshi R Sakuma K . Association of sagittal spinal alignment with thickness and echo intensity of lumbar back muscles in middle-aged and elderly women. Arch Gerontology Geriatrics (2015) 61(2):197–201. doi: 10.1016/j.archger.2015.05.010 26058723

[9] Noonan AM Brown SHM . Paraspinal muscle pathophysiology associated with low back pain and spine degenerative disorders. JOR Spine (2021) 4(3):e1171. doi: 10.1002/jsp2.1171 34611593 PMC8479522

[10] Cho TG Park SW Kim YB . Chronic paraspinal muscle injury model in rat. J Kor Neurosurgical Soc (2016) 59(5):430–6. doi: 10.3340/jkns.2016.59.5.430 PMC5028601 27651859

[11] Hey HWD Lam WMR Chan CX Zhuo WH Crombie EM Tan TC . Paraspinal myopathy-induced intervertebral disc degeneration and thoracolumbar kyphosis in Tsc1mko mice model-a preliminary study. Spine J Off J North Am Spine Soc (2022) 22(3):483–94. doi: 10.1016/j.spinee.2021.09.003 34653636

[12] Özcan-Ekşi EE Ekşi M Turgut VU Canbolat Ç Pamir MN . Reciprocal relationship between multifidus and psoas at L4-L5 level in women with low back pain. Br J Neurosurg (2021) 35(2):220–8. doi: 10.1080/02688697.2020.1783434 32576034

[13] Kalichman L Carmeli E Been E . The association between imaging parameters of the paraspinal muscles, spinal degeneration, and low back pain. BioMed Res Int (2017) 2017:2562957. doi: 10.1155/2017/2562957 28409152 PMC5376928

[14] Sayson JV Hargens AR . Pathophysiology of low back pain during exposure to microgravity. Aviation space Environ Med (2008) 79(4):365–73. doi: 10.3357/asem.1994.2008 18457293

[15] Hides JA Lambrecht G Sexton CT Pruett C Petersen N Jaekel P . The effects of exposure to microgravity and reconditioning of the lumbar multifidus and anterolateral abdominal muscles: Implications for people with lbp. Spine journal: Off J North Am Spine Soc (2021) 21(3):477–91. doi: 10.1016/j.spinee.2020.09.006 32966906

[16] Bailey JF Nyayapati P Johnson GTA Dziesinski L Scheffler AW Crawford R . Biomechanical changes in the lumbar spine following spaceflight and factors associated with postspaceflight disc herniation. Spine journal: Off J North Am Spine Soc (2022) 22(2):197–206. doi: 10.1016/j.spinee.2021.07.021 34343665

[17] Wang X Jia R Li J Zhu Y Liu H Wang W . Research progress on the mechanism of lumbarmultifidus injury and degeneration. Oxid Med Cell Longevity (2021) 2021:6629037. doi: 10.1155/2021/6629037 PMC7936897 33728023

[18] Shahidi B Hubbard JC Gibbons MC Ruoss S Zlomislic V Allen RT . Lumbar multifidus muscle degenerates in individuals with chronic degenerative lumbar spine pathology. J orthopaedic research: Off Publ Orthopaedic Res Soc (2017) 35(12):2700–6. doi: 10.1002/jor.23597 PMC5677570 28480978

[19] Kudo D Miyakoshi N Hongo M Kasukawa Y Ishikawa Y Fujii M . Mrna expressions of peroxisome proliferator-activated receptor gamma coactivator 1α, tumor necrosis factor-A, and interleukin-6 in paraspinal muscles of patients with lumbar kyphosis: A preliminary study. Clin Interventions Aging (2018) 13:1633–8. doi: 10.2147/cia.S172952 PMC6135076 30233161

[20] Shahidi B Fisch KM Gibbons MC Ward SR . Increased fibrogenic gene expression in multifidus muscles of patients with chronic versus acute lumbar spine pathology. Spine (Phila Pa 1976) (2020) 45(4):E189–e95. doi: 10.1097/brs.0000000000003243 PMC6994378 31513095

[21] Lerer A Nykamp SG Harriss AB Gibson TW Koch TG Brown SH . Mri-based relationships between spine pathology, intervertebral disc degeneration, and muscle fatty infiltration in chondrodystrophic and non-chondrodystrophic dogs. Spine journal: Off J North Am Spine Soc (2015) 15(11):2433–9. doi: 10.1016/j.spinee.2015.08.014 26282102

[22] Olaso-Gonzalez G Ferrando B Derbre F Salvador-Pascual A Cabo H Pareja-Galeano H . Redox regulation of E3 ubiquitin ligases and their role in skeletal muscle atrophy. Free Radical Biol Med (2014) 75 Suppl 1:S43–4. doi: 10.1016/j.freeradbiomed.2014.10.799 26461377

[23] Yang JE Zhao KH Qu Y Zou YC . Increased serum Cxcl10 levels are associated with clinical severity and radiographic progression in patients with lumbar disc degeneration. Clinica chimica acta; Int J Clin Chem (2022) 525:15–22. doi: 10.1016/j.cca.2021.12.006 34902344

[24] Yamakuchi M Higuchi I Masuda S Ohira Y Kubo T Kato Y . Type I muscle atrophy caused by microgravity-induced decrease of myocyte enhancer factor 2c (Mef2c) protein expression. FEBS Lett (2000) 477(1-2):135–40. doi: 10.1016/s0014-5793(00)01715-4 10899324

[25] Mirzoev TM Vil'chinskaia NA Lomonosova Iu N Nemirovskaia TL Shenkman BS . [Effect of 30-day space flight and subsequent readaptation on the signaling processes in m. longissimus dorsi of mice]. Aviakosmicheskaia i ekologicheskaia meditsina = Aerospace Environ Med (2014) 48(2):12–5. 25087406

[26] Ogneva IV Maximova MV Larina IM . Structure of cortical cytoskeleton in fibers of mouse muscle cells after being exposed to a 30-day space flight on board the bion-M1 biosatellite. J Appl Physiol (Bethesda Md: 1985) (2014) 116(10):1315–23. doi: 10.1152/japplphysiol.00134.2014 24674857

[27] Gambara G Salanova M Ciciliot S Furlan S Gutsmann M Schiffl G . Microgravity-induced transcriptome adaptation in mouse paraspinal longissimus dorsi muscle highlights insulin resistance-linked genes. Front Physiol (2017) 8:279. doi: 10.3389/fphys.2017.00279 28529490 PMC5418220

[28] Barrett T Wilhite SE Ledoux P Evangelista C Kim IF Tomashevsky M . Ncbi geo: Archive for functional genomics data sets–update. Nucleic Acids Res (2013) 41:D991–5. doi: 10.1093/nar/gks1193 PMC3531084 23193258

[29] Davis S Meltzer PS . Geoquery: A bridge between the gene expression omnibus (Geo) and bioconductor. Bioinf (Oxford England) (2007) 23(14):1846–7. doi: 10.1093/bioinformatics/btm254 17496320

[30] Wu T Hu E Xu S Chen M Guo P Dai Z . Clusterprofiler 4.0: A universal enrichment tool for interpreting omics data. Innovation (Cambridge (Mass) (2021) 2(3):100141. doi: 10.1016/j.xinn.2021.100141 34557778 PMC8454663

[31] Gu Z Eils R Schlesner M . Complex heatmaps reveal patterns and correlations in multidimensional genomic data. Bioinf (Oxford England) (2016) 32(18):2847–9. doi: 10.1093/bioinformatics/btw313 27207943

[32] Zhou N Bao J . Ferrdb: A manually curated resource for regulators and markers of ferroptosis and ferroptosis-disease associations. Database: J Biol Database Curation (2020) 2020:1–8. doi: 10.1093/database/baaa021 PMC7100629 32219413

[33] Liberzon A Birger C Thorvaldsdóttir H Ghandi M Mesirov JP Tamayo P . The molecular signatures database (Msigdb) hallmark gene set collection. Cell Syst (2015) 1(6):417–25. doi: 10.1016/j.cels.2015.12.004 PMC4707969 26771021

[34] Szklarczyk D Gable AL Lyon D Junge A Wyder S Huerta-Cepas J . String V11: Protein-protein association networks with increased coverage, supporting functional discovery in genome-wide experimental datasets. Nucleic Acids Res (2019) 47(D1):D607–d13. doi: 10.1093/nar/gky1131 PMC6323986 30476243

[35] Dixon SJ Lemberg KM Lamprecht MR Skouta R Zaitsev EM Gleason CE . Ferroptosis: An iron-dependent form of nonapoptotic cell death. Cell (2012) 149(5):1060–72. doi: 10.1016/j.cell.2012.03.042 PMC3367386 22632970

[36] Anderson B Ordaz A Zlomislic V Allen RT Garfin SR Schuepbach R . Paraspinal muscle health is related to fibrogenic, adipogenic, and myogenic gene expression in patients with lumbar spine pathology. BMC musculoskeletal Disord (2022) 23(1):608. doi: 10.1186/s12891-022-05572-7 PMC9229083 35739523

[37] Kumar A Tahimic CGT Almeida EAC Globus RK . Spaceflight modulates the expression of key oxidative stress and cell cycle related genes in heart. Int J Mol Sci (2021) 22(16):9088. doi: 10.3390/ijms22169088 34445793 PMC8396460

[38] Bodine SC Baehr LM . Skeletal muscle atrophy and the E3 ubiquitin ligases Murf1 and Mafbx/Atrogin-1. Am J Physiol Endocrinol Metab (2014) 307(6):E469–84. doi: 10.1152/ajpendo.00204.2014 PMC4166716 25096180

[39] Mahmassani ZS Reidy PT McKenzie AI Stubben C Howard MT Drummond MJ . Age-dependent skeletal muscle transcriptome response to bed rest-induced atrophy. J Appl Physiol (Bethesda Md 1985) (2019) 126(4):894–902. doi: 10.1152/japplphysiol.00811.2018 PMC6485685 30605403

[40] Bongers KS Fox DK Kunkel SD Stebounova LV Murry DJ Pufall MA . Spermine oxidase maintains basal skeletal muscle gene expression and fiber size and is strongly repressed by conditions that cause skeletal muscle atrophy. Am J Physiol Endocrinol Metab (2015) 308(2):E144–58. doi: 10.1152/ajpendo.00472.2014 PMC4297781 25406264

[41] Fox DK Ebert SM Bongers KS Dyle MC Bullard SA Dierdorff JM . P53 and Atf4 mediate distinct and additive pathways to skeletal muscle atrophy during limb immobilization. Am J Physiol Endocrinol Metab (2014) 307(3):E245–61. doi: 10.1152/ajpendo.00010.2014 PMC4121573 24895282

[42] Faust HJ Zhang H Han J Wolf MT Jeon OH Sadtler K . Il-17 and immunologically induced senescence regulate response to injury in osteoarthritis. J Clin Invest (2020) 130(10):5493–507. doi: 10.1172/jci134091 PMC7524483 32955487

[43] Segalés J Perdiguero E Serrano AL Sousa-Victor P Ortet L Jardí M . Sestrin prevents atrophy of disused and aging muscles by integrating anabolic and catabolic signals. Nat Commun (2020) 11(1):189. doi: 10.1038/s41467-019-13832-9 31929511 PMC6955241

[44] Li S Wu B Ling Y Guo M Qin B Ren X . Epigenetic landscapes of single-cell chromatin accessibility and transcriptomic immune profiles of T cells in covid-19 patients. Front Immunol (2021) 12:625881. doi: 10.3389/fimmu.2021.625881 33717140 PMC7943924

[45] Liu TT Luo R Yang Y Cheng YC Chang D Dai W . Lrg1 mitigates renal interstitial fibrosis through alleviating capillary rarefaction and inhibiting inflammatory and pro-fibrotic cytokines. Am J Nephrol (2021) 52(3):228–38. doi: 10.1159/000514167 33823527

[46] Frempah B Luckett-Chastain LR Calhoun KN Gallucci RM . Keratinocyte-specific deletion of the il-6rα exacerbates the inflammatory response during irritant contact dermatitis. Toxicology (2019) 423:123–31. doi: 10.1016/j.tox.2019.05.015 31158415

[47] Wang S Yu H Gao J Chen J He P Zhong H . Palmd regulates aortic valve calcification *Via* altered glycolysis and nf-Kb-Mediated inflammation. J Biol Chem (2022) 298(5):101887. doi: 10.1016/j.jbc.2022.101887 35367413 PMC9065630

[48] Zhao D Deng SC Ma Y Hao YH Jia ZH . Mir-221 alleviates the inflammatory response and cell apoptosis of neuronal cell through targeting Tnfaip2 in spinal cord ischemia-reperfusion. Neuroreport (2018) 29(8):655–60. doi: 10.1097/wnr.0000000000001013 29596155

[49] Saban R D'Andrea MR Andrade-Gordon P Derian CK Dozmorov I Ihnat MA . Regulatory network of inflammation downstream of proteinase-activated receptors. BMC Physiol (2007) 7:3. doi: 10.1186/1472-6793-7-3 17397547 PMC1853107

[50] Choi EB Jeong JH Jang HM Ahn YJ Kim KH An HS . Skeletal lipocalin-2 is associated with iron-related oxidative stress in Ob/Ob mice with sarcopenia. Antioxidants (Basel Switzerland) (2021) 10(5):758. doi: 10.3390/antiox10050758 PMC8150392 34064680

[51] Keping Y Yunfeng S Pengzhuo X Liang L Chenhong X Jinghua M . Sestrin1 inhibits oxidized low-density lipoprotein-induced activation of Nlrp3 inflammasome in macrophages in a murine atherosclerosis model. Eur J Immunol (2020) 50(8):1154–66. doi: 10.1002/eji.201948427 32297666

[52] Li YP Chen Y Li AS Reid MB . Hydrogen peroxide stimulates ubiquitin-conjugating activity and expression of genes for specific E2 and E3 proteins in skeletal muscle myotubes. Am J Physiol.-Cell Physiol (2003) 285:C806–12. doi: 10.1152/ajpcell.00129.2003 12773310

[53] Powers SK Smuder AJ Criswell DS . Mechanistic links between oxidative stress and disuse muscle atrophy. Antioxid Redox Signal (2011) 15:2519–28. doi: 10.1089/ars.2011.3973 PMC3208252 21457104

[54] Smuder AJ Kavazis AN Hudson MB Nelson WB Powers SK . Oxidation enhances myofibrillar protein degradation *via* calpain and caspase-3. Free Radic Biol Med (2010) 49:1152–60. doi: 10.1016/j.freeradbiomed.2010.06.025 PMC2930052 20600829

[55] Sun G Xue R Yao F Liu D Huang H Chen C . The critical role of sestrin 1 in regulating the proliferation of cardiac fibroblasts. Arch Biochem Biophys (2014) 542:1–6. doi: 10.1016/j.abb.2013.11.011 24315959

[56] Song E Ramos SV Huang X Liu Y Botta A Sung HK . Holo-lipocalin-2-derived siderophores increase mitochondrial ROS and impair oxidative phosphorylation in rat cardiomyocytes. Proc Natl Acad Sci U S A (2018) 115(7):1576–81. doi: 10.1073/pnas.1720570115 PMC5816208 29378951

