# Supplementary material for: Identification of differentially expressed genes in mouse paraspinal muscle in response to microgravity
Source: Front Endocrinol (Lausanne). 2022 Oct 13;13:1020743. doi: 10.3389/fendo.2022.1020743 (PMC9611771; doi:10.3389/fendo.2022.1020743)
Supplement: Supplementary Table 1 — Detailed information on ferroptosis-related genes. [file DataSheet_1.pdf]

FRGs
AKR1C2
AKR1C3
RB1
HSF1
GCLC
SQSTM1
NQO1
MUC1
MT1G
CISD1
FANCD2
FTMT
HSPA5
HELLS
SCD
SRC
STAT3
PML
MTOR
NFS1
TP63
CDKN1A
MIR137
ENPP2
FH
CISD2
MIR9-1
MIR9-2
MIR9-3
ISCU
ACSL3
OTUB1
CD44
LINC00336
BRD4
PRDX6
MIR17
NF2
ARNTL
JUN
CA9
TMBIM4
PLIN2
MIR212
Fer1HCH
AIFM2
LAMP2
ZFP36
PROM2
CHMP5
CHMP6
CAV1
GCH1

DAZAP1
PIR
HCAR1
SLC16A1
NR4A1
RPTOR
SREBF1
SREBF2
FZD7
P4HB
NT5DC2
BCAT2
PLA2G6
MIR424
PARK7
FXN
SUV39H1
ATF2
ACOT1
ALDH3A2
STK11
FNDC5
CircIL4R
CDH1
NEDD4L
BRD2
BRD3
BRDT
DECR1
GLRX5
NCOA3
NR5A2
PANX2
RHEBP1
TFAP2A
CP
ARF6
ABHD12
PPP1R13L
TFAM
KDM3B
RNF113A
AHCY
circ-TTBK2
MIR522
IDH2
PPARA
SIAH2
VDR
NEDD4
AR
MTF1
COPZ1
NUPR1

USP35
NEAT1
PARP1
PARP2
PARP3
PARP4
PARP6
PARP8
PARP9
PARP10
PARP11
PARP12
PARP14
PARP15
PARP16
PDSS2
TXN
SENP1
OIP5-AS1
MIR190A
FGF21
CREB1
CREB3
CREB5
MIR130B
BEX1
ASAH2
FABP4
AKT1S1
MLST8
TYRO3
SIRT6
TMSB4X
TMSB4Y
KIF20A
ECH1
circRHOT1
ETV4
MEG8
VCP
circ_0007142
RBMS1
KDM4A
MGST1
circKIF4A
miR-7-5p
circ_0067934
MPC1
CHMP1A
CAMKK2
SOX2
SRSF9
PROK2
MIR4443

SIRT2
circRNA1615
MIR27A
MIR670
MEF2C
EZH2
PEDS1
ADAMTS13
CDC25A
SFRS9
CAV
CircFNDC3B
PPARD
ENO3
LCN2
MARCHF5
TRIB2
DHODH
MIR545
PDK4
CircPVT1
MIR9-3HG
ADIPOQ
circDTL
mmu_circRNA_0000309
PTPN18
ABCC5
CISD3
MS4A15
FURIN
circRHBG
GALNT14
KLHDC3
LINC01833
circGFRA1
MAPKAP1
PRR5
RICTOR
GSTM1
TERT
circ0097009
TMEM161B-DT
circEPSTI1
MIR18A
RARRES2
HSPB1
SLC40A1
GPX4
NFE2L2
FTH1
SLC3A2
CBS
SESN2
FTL

RRM2
GDF15
NOS2
RELA
PRDX1
DUSP1
NCF2
MT3
UBC
ALB
TXNRD1
SRXN1
GPX2
BNIP3
OXSR1
SELENOS
ANGPTL7
DDIT4
LOC284561
ASNS
TSC22D3
DDIT3
JDP2
SLC1A4
PCK2
TXNIP
VLDLR
GPT2
PSAT1
LURAP1L
SLC7A5
HERPUD1
XBP1
ZNF419
KLHL24
TRIB3
ZFP69B
ATP6V1G2
VEGFA
TUBE1
ARRDC3
CEBPG
SNORA16A
RGS4
BLOC1S5-TXNDC5
LOC390705
EIF2S1
KIM-1
CXCL2
HSD17B11
SETD1B
MAFG
IL33
HAMP

STEAP3
DRD5
DRD4
MAP3K5
SLC2A1
SLC2A3
SLC2A6
SLC2A8
SLC2A12
GLUT13
SLC2A14
EIF2AK4
TFAP2C
SP1
HBA1
NNMT
PLIN4
HIC1
STMN1
CAPG
HNF4A
NGB
YWHAE
GABPB1
AURKA
MIR4715
RIPK1
MIR30B
MMP13
LRRFIP1
CBR1
PTGS2
AKR1C1
RPL8
IREB2
ATP5MC3
CS
EMC2
ACSF2
NOX1
CYBB
NOX3
NOX4
NOX5
DUOX1
DUOX2
G6PD
PGD
VDAC2
PIK3CA
FLT3
SCP2
TP53
ACSL4

LPCAT3
NRAS
KRAS
HRAS
TF
TFRC
TFR2
SLC38A1
SLC1A5
GLS2
GOT1
CARS1
ALOX5
KEAP1
HMOX1
ATG5
ATG7
NCOA4
ALOX12
ALOX12B
ALOX15
ALOX15B
ALOXE3
PHKG2
ACO1
G6PDX
ULK1
ATG3
ATG4D
BECN1
MAP1LC3A
GABARAPL2
GABARAPL1
ATG16L1
WIPI1
WIPI2
SNX4
ATG13
ULK2
SAT1
EGFR
MAPK3
MAPK1
BID
ZEB1
DPP4
CDKN2A
PEBP1
SOCS1
CDO1
MYB
MAPK8
MAPK9
CHAC1

MAPK14
LINC00472
PRKAA2
PRKAA1
ELAVL1
BAP1
ABCC1
MIR6852
ACVR1B
TGFBR1
EPAS1
HILPDA
HIF1A
IFNG
ANO6
LPIN1
HMGB1
TNFAIP3
TLR4
ATF3
ATM
YY1AP1
EGLN2
MIOX
TAZ
MTDH
IDH1
SIRT1
FBXW7
PANX1
DNAJB6
BACH1
LONP1
CD82
IL1B
CTSB
POR
CYB5R1
ELOVL5
FADS1
FBW7
PTEN
NR1D1
NR1D2
TBK1
IL6
USP7
miR-182-5p
miR-378a-3p
ATF4
AQP3
AQP5
AQP8
LINC00618

MT1DP
PEX10
AGPAT3
PEX12
CHP1
GPAT4
BRPF1
OSBPL9
INTS2
MMD
CYP4F8
MLLT1
TTPA
GRIA3
EPT1
POM121L12
LIG3
AEBP2
AGPS
CDCA3
PEX2
PEX6
TIMM9
DCAF7
LCE2C
FAR1
PHF21A
SMAD7
LYRM1
AMN
PEX3
MTCH1
ACADSB
PVT1
hsa_circ_0008367
SLC39A14
MAP3K11
GSK3B
BRD7
SLC25A28
MFN2
SLC11A2
ZFAS1
TSC1
TGFB1
SNCA
SIRT3
CGAS
STING1
HDDC3
MIR761
MDM2
MDM4
MIR214

DLD
WWTR1
PRKCA
LGMN
SMPD1
MYCN
IFNA1
IFNA2
IFNA4
IFNA5
IFNA6
IFNA7
IFNA8
IFNA10
IFNA13
IFNA14
IFNA16
IFNA17
IFNA21
SMG9
PPARG
miR-335
SNX5
PAQR3
MICU1
TOR2A
MIR375
MAP3K14
CircKDM4C
MIR324
QSOX1
MIB2
CLTRN
KLF2
MIR5096
HOTAIR
H19
FOXO4
YTHDC2
DDR2
SLC39A7
TRIM46
ACSL1
KDM5A
TRIM21
DPEP1
CYGB
IDO1
GSTZ1
GJA1
SLC7A11
PGRMC1
CIRBP
circPSEN1

USP11
YAP
MIR135B
TRIM26
NDRG1
MIR302A
ASMTL-AS1
FADS2
PIEZO1
LIFR
PTPN6
MIR15A
EGR1
ADAM23
ARHGEF26-AS1
CPEB1
COX4I2
lncRNA AABR07017145.1
TIMP1
KDM6B
METTL14
MIB1
KDM5C
MEG3
CCDC6
CFL1
MIR539
KMT2D
