# Supplementary material for: Identification of differentially expressed genes in mouse paraspinal muscle in response to microgravity
Source: Front Endocrinol (Lausanne). 2022 Oct 13;13:1020743. doi: 10.3389/fendo.2022.1020743 (PMC9611771; doi:10.3389/fendo.2022.1020743)
Supplement: Supplementary Table 2 — Detailed information on oxidative stress-related genes. [file DataSheet_2.pdf]

OSRGs
ABCD1
ABL1
ACOX2
ADA
ADAM9
ADIPOQ
ABCC2
ADNP2
ADPRS
AGAP3
AIF1
AIFM2
AKR1C3
AKT1
ALAD
ALDH3B1
ALOX5
ANGPTL7
ANKRD2
ANKZF1
ANXA1
APEX1
APOA4
APOD
APOE
APP
APTX
AQP1
ARG1
ARL6IP5
ARNT
ARNTL
ATF4
ATG7
ATOX1
ATP13A2
ATP2A2
ATP7A
ATRN
AXL
BAD
BAG5
BAK1
BCL2
BECN1
BMP7
BNIP3
BRF2
BTK
C19orf12
CA3
CAMKK2
CAPN2

CASP3
CAT
CBX8
CCL19
CCNA2
CCR7
CCS
CD36
CD38
CDK2
CFLAR
CHCHD2
CHD6
CHRNA4
CHUK
CLN8
COA8
COL1A1
CPEB2
CRK
CRYAB
CRYGD
CYBA
CYBB
CYCS
CYGB
CYP1B1
CYP2E1
DAPK1
DGKK
DHCR24
DHFR
DHFRP1
DHRS2
DIABLO
DNM2
DPEP1
DUOX1
DUOX2
DUSP1
ECT2
EDN1
EEF2
EGFR
EGLN1
EIF2S1
ENDOG
EPAS1
EPX
ERCC1
ERCC2
ERCC3
ERCC6
ERCC6L2

ERCC8
ERMP1
ERN1
ERO1A
ETFDH
ETS1
ETV5
EZH2
FABP1
FANCC
FANCD2
FBLN5
FBXO7
FBXW7
FER
FGF8
FKBP1B
FOS
FOSL1
FOXO1
FOXO3
FUT8
FXN
FYN
G6PD
GATA4
GCH1
GCLC
GCLM
GGT7
GJB2
GLRX2
GNAO1
GPR37
GPR37L1
GPX1
GPX2
GPX3
GPX4
GPX5
GPX6
GPX7
GPX8
GSKIP
GSR
GSS
GSTP1
GUCY1B1
H19
HAO1
HBA1
HBA2
HBB
HDAC2

HDAC6
HGF
HIF1A
HMOX1
HMOX2
HNRNPD
HNRNPM
HP
HSF1
HSPA1A
HSPA1B
HSPB1
HTRA2
HYAL1
HYAL2
IDH1
IL10
IL18RAP
IL6
IMPACT
INS
IPCEF1
JAK2
JUN
KCNA5
KCNC2
KDM6B
KEAP1
KLF2
KLF4
KRT1
LANCL1
LDHA
LIAS
LONP1
LPO
LRRK2
MACROH2A1
MAP1LC3A
MAP3K5
MAPK1
MAPK13
MAPK3
MAPK7
MAPK8
MAPK9
MAPKAP1
MAPT
MB
MBL2
MCL1
MCTP1
MEAK7
MELK

MET
MGAT3
MGST1
MICB
MIR103A1
MIR107
MIR132
MIR133A1
MIR17
MIR195
MIR19A
MIR21
MIR29B1
MIR34A
MIR675
MIR92A1
MIRLET7B
MMP14
MMP2
MMP3
MMP9
MPO
MPV17
MSRA
MSRB2
MSRB3
MT-CO1
MT-ND1
MT-ND3
MT-ND5
MT-ND6
MT3
MTF1
MTR
MYB
MYEF2
NAPRT
NCF1
NCF2
NCF4
NCOA7
NDUFA12
NDUFA6
NDUFB4
NDUFS2
NDUFS8
NEIL1
NET1
NFE2L1
NFE2L2
NME2
NME5
NME8
NOL3

NONO
NOS3
NOX1
NOX4
NOX5
NQO1
NR4A2
NR4A3
NUDT1
NUDT15
NUDT2
OGG1
OSER1
OXR1
OXSR1
P4HB
PAGE4
PARK7
PARP1
PAWR
PAX2
PCGF2
PCNA
PDCD10
PDE8A
PDGFD
PDGFRA
PDGFRB
PDK1
PDK2
PDLIM1
PENK
PINK1
PJVK
PKD2
PLA2R1
PLEKHA1
PLK3
PML
PNKP
PNPT1
PPARGC1A
PPARGC1B
PPIA
PPIF
PPP1R15B
PPP2CB
PPP5C
PRDX1
PRDX2
PRDX3
PRDX4
PRDX5
PRDX6

PRKAA1
PRKAA2
PRKCD
PRKD1
PRKN
PRKRA
PRNP
PRODH
PRR5L
PSEN1
PSIP1
PSMB5
PTGS1
PTGS2
PTK2B
PTPRK
PTPRN
PXDN
PXDNL
PXN
PYCR1
PYCR2
PYROXD1
RACK1
RAD52
RBM11
RBPMS
RELA
REST
RGS14
RHOB
RIPK1
RIPK3
RNF112
ROMO1
RPS3
S100A7
SCARA3
SCGB1A1
SDC1
SELENOK
SELENON
SELENOP
SELENOS
SESN1
SESN2
SESN3
SETX
SFPQ
SGK2
SIGMAR1
SIN3A
SIRPA
SIRT1

SIRT2
SLC1A1
SLC23A2
SLC25A24
SLC7A11
SLC8A1
SMPD3
SNCA
SOD1
SOD2
SOD3
SP1
SPHK1
SRC
SRXN1
STAR
STAU1
STK24
STK25
STK26
STOX1
STX2
STX4
SUMO4
TAT
TBC1D24
THG1L
TLDC2
TLR4
TLR6
TMEM161A
TNFAIP3
TOR1A
TP53
TP53INP1
TPM1
TPO
TRA2B
TRAF2
TRAP1
TREX1
TRPA1
TRPC6
TRPM2
TSC1
TXN
TXN2
TXNIP
TXNRD1
TXNRD2
UBE3A
UBQLN1
UCN
UCP1

UCP2
UCP3
VKORC1L1
VNN1
VRK2
WNT1
WNT16
WRN
XRCC1
ZC3H12A
ZNF277
ZNF580
ZNF622
